# Diagnosis of canine spontaneous hypoadrenocorticism

**DOI:** 10.1186/s40575-022-00119-4

**Published:** 2022-05-03

**Authors:** Pedro J. Guzmán Ramos, Michael Bennaim, Robert E. Shiel, Carmel T. Mooney

**Affiliations:** 1 University College Dublin Veterinary Hospital, University College Dublin, Dublin, Ireland; 2 Centre Hospitalier Vétérinaire Anicura Aquivet, Eysines, France

**Keywords:** Adrenals, Cortisol, Aldosterone, Addison´s, Endocrinology

## Abstract

Hypoadrenocorticism is characterized by a reduction in mineralocorticoid and/or glucocorticoid production by the adrenal glands. Several subtypes have been described with different clinical and clinicopathological consequences. Most affected dogs have vague and non-specific signs that precede an eventual life-threatening crisis. This review aims to appraise classification, the available data on epidemiology and the clinical and laboratory features of naturally occurring canine hypoadrenocorticism.

## Plain English Summary

Canine hypoadrenocorticism is a relatively uncommon endocrine disease that can present with a wide variety of clinical signs resulting from cortisol or aldosterone deficiency or both. Hypoadrenocorticism should be considered in all dogs with severe illness and typical electrolyte abnormalities but also in those with waxing and waning clinical signs. Multiple clinical and laboratory features are suggestive of the disease and should prompt evaluation of adrenal function. The ACTH stimulation test is the best test for diagnosing hypoadrenocorticism but, in those cases without the typical presentation, evaluation of aldosterone secretory capacity and endogenous ACTH concentrations should be performed to distinguish primary from secondary disease. In this review we discuss the pathophysiology of the disease, the clinical signs and laboratory features that should raise suspicion of hypoadrenocorticism and the performance of the different diagnostic tests.

## Introduction

Hypoadrenocorticism is an uncommon endocrine disorder of dogs with an estimated prevalence between 0.06 and 0.28% [1]. When encountered, if it is not appropriately diagnosed and treated, the disease can be associated with significant mortality due to severe hyperkalaemia, hyponatraemia, dehydration and hypovolaemic shock. A high index of suspicion is required to consider hypoadrenocorticism as a differential diagnosis, particularly before treatment is administered that could interfere with diagnostic testing. This review will evaluate the epidemiological, clinical and clinicopathological features of the disease with the aim of helping to raise suspicion for its presence and diagnosis.

### Physiology

**Fig. 1:**
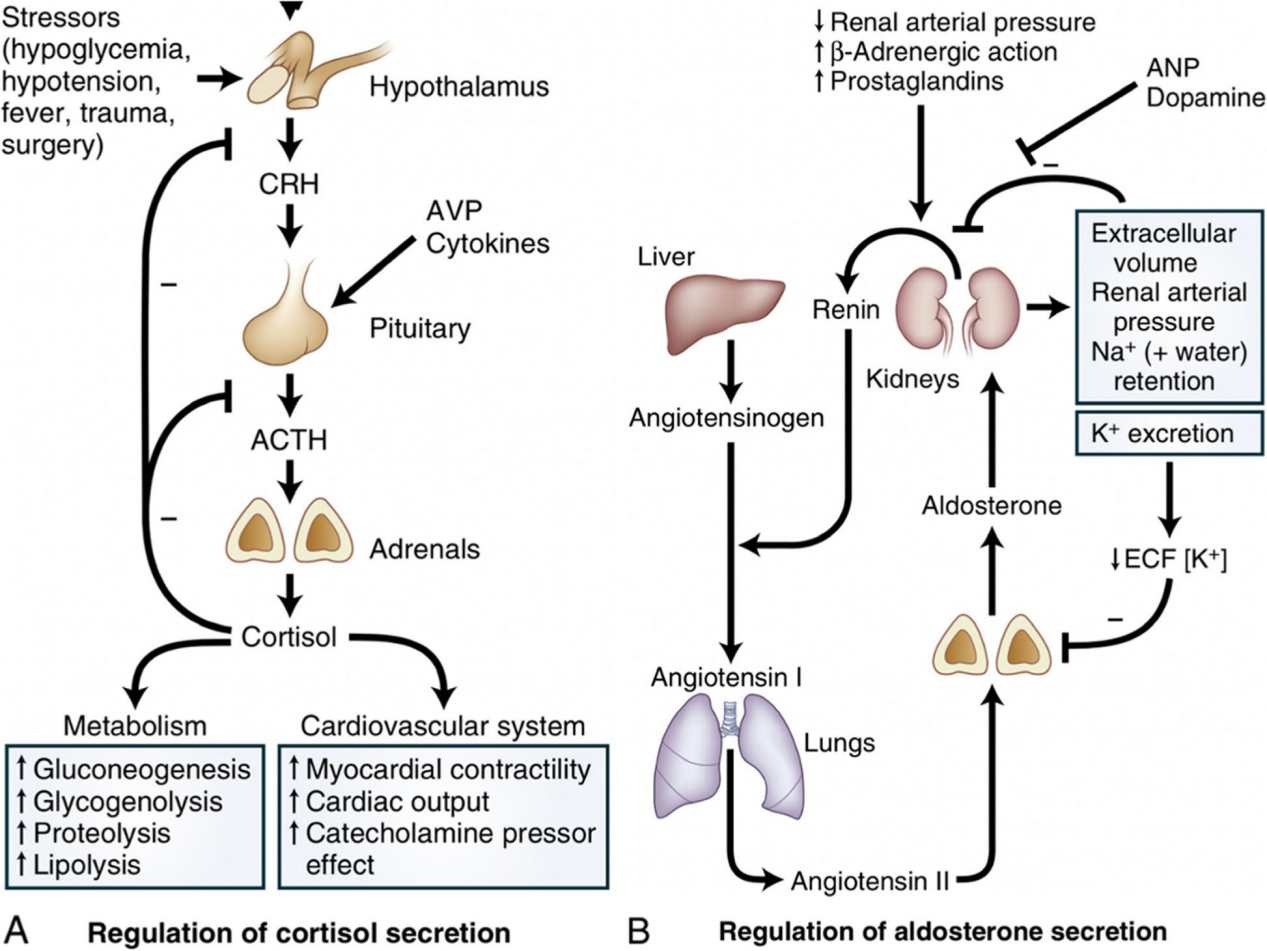
Normal feedback regulation of cortisol and aldosterone secretion. **A** Hypothalamic–pituitary–adrenal axis. Adrenocorticotropic hormone (ACTH) is secreted from the anterior pituitary under the influence of corticotropin releasing hormone (CRH) although other factors such as stress and arginine vasopressin (AVP) may also play a role. Secretion of CRH and ACTH is inhibited by cortisol, highlighting the importance of negative feedback control. **B** Renin–angiotensin–aldosterone system (RAAS). Renin is secreted by the juxtaglomerular cells in the kidney dependent on renal arteriolar blood pressure. Renin converts angiotensinogen to angiotensin I, which is converted in the lungs by angiotensin converting enzyme (ACE) into angiotensin II. Decreasing extracellular fraction of potassium (K^+^) has an important direct inhibitory effect on aldosterone secretion. Reproduced and modified with permission from Newell Price and Auchus, 2020 [2]

The adrenal glands are divided into an outer cortex and inner medulla. The adrenal cortex has three functionally and morphologically distinct zones. The outermost zona glomerulosa is responsible for the production of mineralocorticoids (aldosterone) whilst glucocorticoids (cortisol) and androgens are produced within the inner zona fasciculata and reticularis. The secretion of aldosterone is regulated primarily by circulating potassium and angiotensin II concentrations. Adrenocorticotropic hormone (ACTH) can stimulate aldosterone secretion but to a much lesser extent than either angiotensin II or potassium. By contrast, cortisol production is tightly regulated by corticotropin releasing hormone (CRH) and plasma ACTH concentrations (Fig. 1).

#### Functions of cortisol

Cortisol regulates circulating glucose concentrations by enhancing hepatic gluconeogenesis and glycogenesis, stimulating lipolysis and protein catabolism and reducing peripheral glucose utilization. It plays an important role in calcium and phosphate metabolism by enhancing urinary calcium excretion and reducing vitamin-D mediated intestinal calcium absorption. Cortisol affects water metabolism by suppressing arginine vasopressin (AVP) release, and by decreasing AVP actions on its receptor and the collecting tubules. Within the gastrointestinal tract, cortisol helps maintain normal function of the mucosa, influences digestion and intestinal absorption of nutrients, increases intestinal brush border and mitochondrial enzymes and impacts the intestinal microbiome. Cortisol helps sustain myocardial performance and increases cardiac output. It facilitates normal responsiveness of arterioles to the constrictive actions of catecholamines and angiotensin II and decreases production of vasodilator prostaglandins to aid maintenance of normal blood pressure and volume. Cortisol stimulates production and decreases destruction of red blood cells and stimulates the release of platelets and neutrophils from the bone marrow while reducing their removal from the circulation. Cortisol also suppresses lymphocyte proliferation and reduces the number of circulating lymphocytes, eosinophils and basophils through peripheral redistribution.

#### Functions of aldosterone

Aldosterone regulates water, acid–base and electrolyte homeostasis. It increases absorption of sodium and excretion of potassium by the kidneys, sweat glands, salivary glands and intestinal epithelial cells. Aldosterone also stimulates potassium uptake by muscle, liver, adipose and nerve tissues. Its main site of action in the kidney is the distal nephron (principal cells of the connecting segment and collecting tubules) where it promotes tubular reabsorption of sodium by stimulating insertion of epithelial sodium channels in the luminal surface, and sodium–potassium ATP-ase pumps in the basolateral membrane. It also increases the number of potassium channels in the luminal membrane which enhances potassium secretion to the luminal fluid. Additionally, aldosterone causes secretion of hydrogen ions in exchange for sodium in the intercalated cells and cortical collecting tubules.

### Causes of hypoadrenocorticism

Primary hypoadrenocorticism refers to any disease resulting in adrenocortical injury that ultimately decreases hormone production. Secondary and tertiary hypoadrenocorticism refer to diseases associated with a deficiency of pituitary ACTH and hypothalamic CRH, respectively. It can be difficult to distinguish secondary and tertiary disease, and the term central hypoadrenocorticism is often used to circumvent this issue.

Immune-mediated destruction is the most common cause of primary hypoadrenocorticism in dogs and it has a strong genetic association in some breeds. Less common causes, reported in individual cases, include neoplasia, tuberculosis, fungal disease, other granulomatous or infectious diseases, infarcts and drugs (particularly trilostane or mitotane) [3–5]. Secondary hypoadrenocorticism can result from any vascular, inflammatory, infectious, traumatic, developmental or neoplastic process involving the hypophysis. It is inevitable after surgical hypophysectomy and can result, albeit temporarily, from prior glucocorticoid or progestin administration [6–11]. A few cases of idiopathic secondary hypoadrenocorticism have been reported. Tertiary hypoadrenocorticism has not yet been definitively diagnosed in dogs.

Most dogs with primary hypoadrenocorticism have both glucocorticoid and mineralocorticoid deficiency, and consequently present with associated electrolyte disturbances. However, glucocorticoid deficiency can occur without apparent concurrent mineralocorticoid deficiency. This is expected in dogs with central hypoadrenocorticism but can also be seen in some dogs with primary disease [12–15]. The reasons for dogs presenting with primary hypoadrenocorticism without electrolyte abnormalities are varied. Firstly, selective destruction of the zonae fasciculata and reticularis with relative sparing of the zona glomerulosa has been demonstrated histopathologically in some dogs with isolated cortisol deficiency [16, 17]. Secondly, some dogs with demonstrable mineralocorticoid deficiency maintain electrolyte concentrations within reference interval through an as of yet unknown mechanism [15]. Lastly, symptomatic treatment, such as intravenous fluid therapy, may correct electrolyte derangements prior to presentation for further evaluation [18]. Some dogs with primary hypoadrenocorticism and reference interval electrolyte concentrations eventually progress to develop hyponatraemia or hyperkalaemia [14].

In some cases of secondary disease, depending on cause, additional hormone deficiencies are described, such as thyroid-stimulating hormone (TSH) or vasopressin [6–11]. There have also been isolated reports of mineralocorticoid deficiency without concurrent glucocorticoid deficiency, although complete investigations were not performed in all and hyporeninaemic hypoaldosteronism was not ruled out in most cases [19–22].

### Terminology

**Fig. 2:**
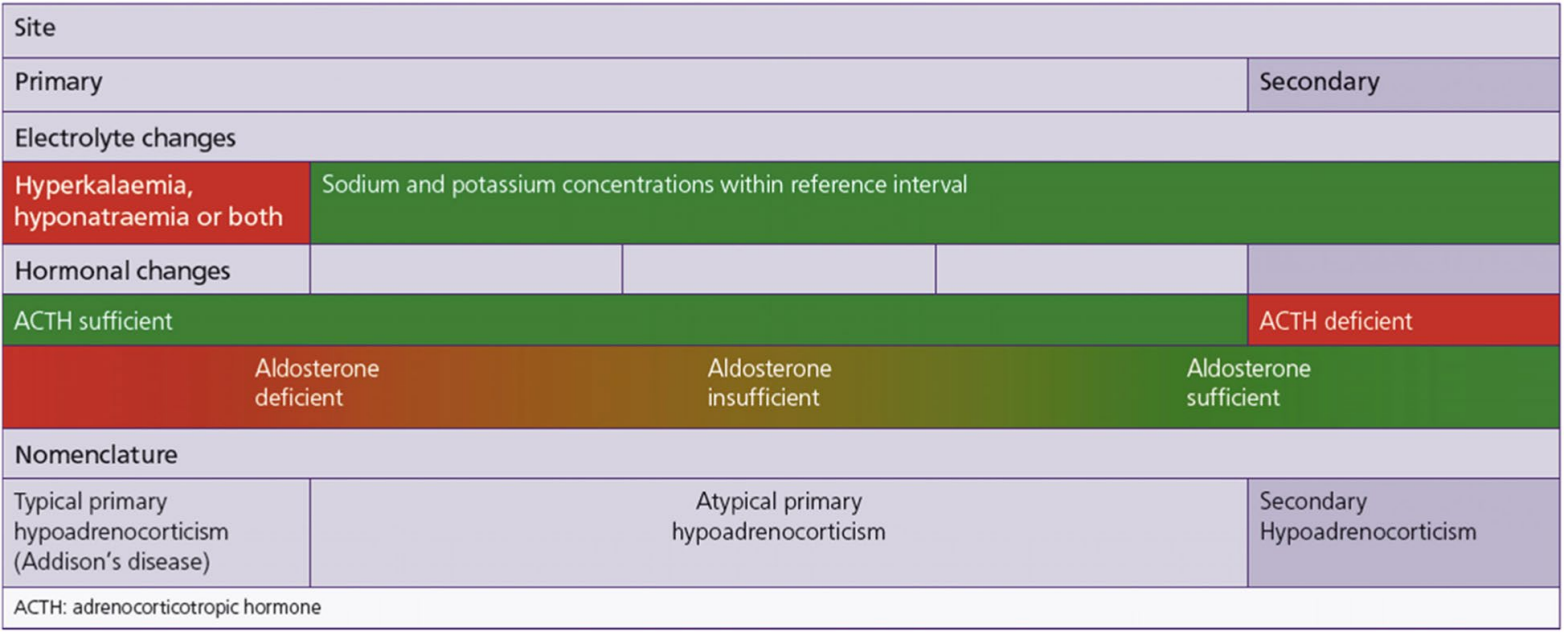
Expected electrolyte and hormonal abnormalities in dogs with hypoadrenocorticism. Apparent isolated glucocorticoid deficiency (with reference interval electrolyte concentrations) can occur with sufficient, insufficient and deficient aldosterone production. Reproduced with permission of UK-Vet Companion Animal [24]

A range of descriptive terms are used to classify the different types of hypoadrenocorticism. Primary hypoadrenocorticism is referred to as ‘typical’ when describing glucocorticoid deficiency combined with hyponatraemia, hyperkalaemia, or both that are induced by mineralocorticoid deficiency. This condition is also known as Addison’s disease. Hypoadrenocorticism referred to as ‘atypical’ loosely describes the presence of glucocorticoid deficiency in the absence of the electrolyte changes expected with hypoaldosteronism. More recently, the European Society for Veterinary Endocrinology (ESVE) established project (Agreeing Language in Veterinary Endocrinology (ALIVE)) has suggested that ‘typical’ and ‘atypical’ disease be referred to as hyponatraemic and/or hyperkalaemic primary hypoadrenocorticism and eunatraemic, eukalaemic primary or secondary hypoadrenocorticism, respectively. However, such a description does not fully describe the fact that reference interval electrolyte concentrations can occur in the face of mineralocorticoid deficiency. Nor does it account for abnormal electrolyte concentrations that can occur with glucocorticoid deficiency alone. Hyponatraemia has been described in humans with isolated glucocorticoid deficiency alone and whilst uncommon, it has been reported in two of five dogs with secondary hypoadrenocorticism where mineralocorticoid deficiency is not expected [18, 23]. Ideally, definitions would be based on both pathophysiological mechanisms and proven hormone status. Primary hypoadrenocorticism should be classified as typical (Addison’s disease) if there is glucocorticoid deficiency together with supportive electrolyte changes of mineralocorticoid deficiency. Further investigation is advised in animals with reference interval electrolyte concentrations to determine if primary or central disease is present. If primary disease is confirmed, additional tests should be performed to assess aldosterone secretory capacity in order to guide treatment and prognosis. The expected hormonal and electrolyte abnormalities associated with the different types of hypoadrenocorticism are illustrated in Fig. 2.

In many case series thus far reported, only glucocorticoid deficiency is definitively diagnosed. Distinction between primary and central disease or evaluation of mineralocorticoid production is often not completed if electrolyte concentrations were not abnormal. For the purposes of this review, hypoadrenocorticism refers to all such cases without further distinction, Addison’s disease if electrolyte abnormalities are present and glucocorticoid deficiency if concomitant with reference interval electrolyte concentrations. If further differentiation is available this will be highlighted.

### Signalment

Hypoadrenocorticism has been reported in dogs aged from 2 months to 14 years, with a median age between 3 and 4 years [18, 25–27]. Dogs diagnosed with glucocorticoid deficiency alone are usually slightly older (typically 6–8 years old) [14, 28, 29].

Hypoadrenocorticism can occur in any cross or pure breed dog but is more common in the latter. It has a strong heritable component in standard poodles, Portuguese water dogs, Nova Scotia duck tolling retrievers, soft coated wheaten terriers and bearded collies [30–38]. Familial hypoadrenocorticism has also been reported in Leonbergers and Pomeranians, where disease prevalence is only increased within certain lines [39, 40]. Several other breeds have been shown to have either increased (e.g., great Dane, Pyrenean mountain dog, West Highland white terrier, Rottweiler) or decreased (e.g., golden retriever, Yorkshire terrier, Lhasa apso) risk of hypoadrenocorticism [18, 26, 41, 42]. Whilst an autosomal recessive mode of inheritance has been suggested in some breeds, a more complex pattern of inheritance as reported in humans is more likely [32, 33, 36, 43]. As a consequence, generalised breeding advice is challenging although it is known that inbreeding increases the risk of hypoadrenocorticism in Portuguese water dogs [36].

The odds of developing hypoadrenocorticism have been described to be between 1.85 to 2.6 times greater in females compared to males in the general dog population [18, 44]. However, such a gender predisposition has not been identified in all breed-specific studies, presumably reflecting different genetic risk factors in individual breeds [30, 32, 33, 36–39].

### Clinical features

A clinical suspicion of hypoadrenocorticism is necessary to prompt investigation for the disease. However, most historical and clinical findings are non-specific and commonly observed in other conditions such as gastrointestinal or kidney disorders. Features that should raise suspicion of hypoadrenocorticism include a waxing and waning course of chronic gastrointestinal signs, particularly if rapidly responsive to symptomatic fluid or glucocorticoid therapy, or where such signs are exacerbated by stressful situations [45]. In some cases, these non-specific signs can quickly progress to an acute crisis characterised by severe dehydration, hypovolaemia, and severe electrolyte and acid–base derangements [41]. Early recognition and treatment of hypoadrenocorticism can prevent these life-threatening consequences. Reported historical and clinical features are summarized in Table 1.

The most common historical features include lethargy along with a variety of gastrointestinal signs, including anorexia, regurgitation, vomiting, diarrhoea and weight loss [14, 18, 25, 27, 37, 38, 42, 46]. Haematemesis and melaena have also been reported, although less commonly. The prominence of gastrointestinal signs is not unexpected because of the physiological role of glucocorticoids in maintenance of gastrointestinal integrity, although their severity may be greater in dogs with concurrent mineralocorticoid deficiency due to the related water deficit, hypovolaemia and hypoperfusion. Dogs with glucocorticoid deficiency alone often remain undiagnosed for longer periods because signs are indistinguishable from primary gastrointestinal disease, and more specific clinical and clinicopathological consequences of mineralocorticoid deficiency are absent [13, 14, 18, 28, 29, 47]. Evaluation of adrenal function should be considered in any dog with chronic, recurrent gastrointestinal signs before performing more invasive tests such as endoscopy and biopsy. Although a recent study described a prevalence of hypoadrenocorticism of only 4% in such cases [48], this is higher than the prevalence of hypoadrenocorticism in the general population. Additionally, the stress of invasive procedures could precipitate development of life-threatening clinical signs in a dog with unrecognised hypoadrenocorticism.

Polyuria and polydipsia are also commonly reported by owners. Polyuria mainly occurs as a result of mineralocorticoid deficiency; sodium wastage results in loss of renal medullary hypertonicity and concentration gradient, which impairs water resorption. Hyponatraemia also interferes with stimulation of arginine vasopressin (AVP) release by reducing serum osmolality [41]. Altered responsiveness to AVP may also occur due to ionised hypercalcaemia, if present. Compensatory polydipsia is common in the initial stages of the disease, but progressive illness can interfere with water intake contributing to dehydration and hypovolaemia [49].

Physical examination findings depend on the severity of the disease, but can include weak pulses, dehydration, hypovolaemia, hypotension, muscle weakness, poor body condition, abdominal pain and, in severe cases, hypovolaemic shock. In severely hypovolaemic cases, the expected tachycardia may be absent because of hyperkalaemia, and bradycardia can also be observed, albeit less commonly. The lack of compensatory tachycardia or bradycardia in a hypovolaemic dog should raise suspicion for hypoadrenocorticism [50]. Hypotension is usually related to the combination of hypovolaemia, lack of compensatory tachycardia and poor vascular tone [49]. One study identified lower median systolic blood pressure in 53 dogs with hypoadrenocorticism compared with 110 dogs with other illnesses (median of 90 and 140 mmHg, respectively) [27].

Other clinical signs reported, although less frequently, include more profound weakness and seizures related to hypoglycaemia, which can be severe [14, 51]. Other comorbidities such as megaoesophagus have been described [14, 28, 52–54]. However, transient oesophageal distension can also be observed in collapsed animals and can be confused with megaoesophagus [55]. The exact aetiopathogenesis of oesophageal dysfunction and distension in hypoadrenocorticism remains unknown. Imbalances in sodium and potassium concentrations could affect membrane potential and neuromuscular function leading to oesophageal distension. However, megaoesophagus has also been described in dogs with suspect glucocorticoid deficiency alone [52–54]. Muscle weakness secondary to cortisol deficiency has been proposed because clinical signs and structural changes usually resolve with glucocorticoid supplementation [41]. However, myasthenia gravis associated with reversible megaoesophagus was not ruled out in many of these cases.

**Table 1:** The prevalence of individual clinical features of both hypoadrenocorticism and atypical hypoadrenocorticism compiling data from the most relevant case series [14, 18, 25, 28, 29, 37, 38, 42, 46]. In most cases of hypoadrenocorticism, mineralocorticoid deficiency was presumed because of associated electrolyte abnormalities but this was not always the case. Some studies did not differentiate primary hypoadrenocorticism and glucocorticoid deficiency alone and clinical features were considered collectively and included in the hypoadrenocorticism group [18, 25, 37, 38]. In most cases of “atypical” hypoadrenocorticism, electrolyte abnormalities were not present and while glucocorticoid deficiency was confirmed, mineralocorticoid production was not usually evaluated. Secondary hypoadrenocorticism was confirmed in only a small number of these cases

Clinical features	**Hypoadrenocorticism**		**“Atypical” hypoadrenocorticism**	
	No. affected (No. evaluated)	%	No. affected (No. evaluated)	%
*Lethargy*	495 (585)	84.6	49 (77)	63.6
*Anorexia*	424 (532)	79.7	37 (77)	48.1
*Vomiting*	420 (585)	71.8	49 (77)	63.6
*Weakness*	226 (450)	50.2	20 (66)	30.3
*Diarrhoea*	222 (532)	41.7	39 (77)	50.6
*Hypothermia*	94 (267)	35.2	0 (77)	0
*Dehydration*	174 (468)	37.2	2 (18)	11.1
*Weight loss*	164 (485)	33.8	21 (77)	27.2
*Abdominal pain*	44 (223)	19.7	9 (77)	11.7
*Polyuria*	86 (460)	18.7	9 (77)	11.7
*Polydipsia*	85 (460)	18.5	10 (77)	12.9
*Melaena*	50 (355)	14.1	4 (59)	6.8
*Haematemesis*	12 (278)	4.3	0 (77)	0
*Shock/collapse*	119 (349)	11.3	3 (29)	10.3
*Incontinence*	3 (35)	8.5	1 (11)	9.1
*Shaking/shivering*	108 (475)	22.7	1 (11)	9.1

*No.* Number

### Clinicopathological features

Relevant clinicopathological features of hypoadrenocorticism are presented in Table 2. The diagnostic performance of different clinicopathological, imaging and endocrine abnormalities are described in Table 3.

#### Haematology

Haematological abnormalities identified in dogs with hypoadrenocorticism include normocytic, normochromic non-regenerative anaemia, relative erythrocytosis, eosinophilia, neutrophilia and lymphocytosis [18, 25].

Anaemia is reported in approximately 30% of cases. This occurs due to lack of stimulation of erythropoiesis related to glucocorticoid deficiency, which results in non-regenerative anaemia. Severe anaemia can also occur due to gastrointestinal haemorrhage. In such cases, at least some regenerative response may be present. Anaemia can be masked by concurrent haemoconcentration, only becoming evident after restoration of circulating volume and hydration status. Anaemia is more commonly reported in dogs with glucocorticoid deficiency alone (up to 60% of cases) likely due to the chronicity of the disease, the high frequency of gastrointestinal signs, and less severe haemoconcentration [14, 28]. Relative erythrocytosis has been reported in approximately 17% of dogs but only those with Addison’s disease, reflecting the more severe volume depletion with this disorder.

A reverse stress leucogram with eosinophilia and lymphocytosis is expected in the absence of cortisol. Cortisol deficiency decreases eosinophil migration into tissues and increases the rate of eosinophil production in the bone marrow resulting in eosinophilia [56] but eosinophilia is present in only approximately 20% of cases. Cortisol has a lymphocytotoxic effect and reduces the number of circulating lymphocytes but lymphocytosis is only reported in 10 to 20% of cases. One study described that a lymphocyte count greater than 5.0 × 10^9^/L had high specificity but low sensitivity for hypoadrenocorticism limiting its diagnostic usefulness [27]. Calculation of the neutrophil to lymphocyte ratio improves diagnostic sensitivity compared to lymphocyte count alone, but this is at the expense of specificity [27, 57]. Thus, eosinophilia and lymphocytosis, although important features of hypoadrenocorticism, are not present in a significant proportion of cases. Nevertheless, the absence of a stress response (neutrophilia, lymphopenia, monocytosis and eosinopenia) despite the presence of systemic illness is inappropriate and should also prompt consideration of hypoadrenocorticism.

#### Serum biochemistry

The most common biochemical abnormalities in dogs with hypoadrenocorticism (> 80% of cases) include hyperkalaemia, hyponatraemia and hypochloraemia [18, 25]. Similar electrolyte changes are observed in dogs with severe gastrointestinal (including intestinal parasitism), kidney and liver disease, congestive heart failure, pleural effusion, severe metabolic or respiratory acidosis and widespread tissue destruction. Evaluation of the Na:K ratio may be a useful tool in differentiating hypoadrenocorticism from these other diseases. The lower the Na:K ratio, the higher the diagnostic specificity for hypoadrenocorticism [26, 27]. Combining the Na:K ratio and lymphocyte count can provide even more information compared to either parameter alone [27, 57]. Clearly these electrolyte abnormalities do not occur with cortisol deficiency alone and are not present in a small proportion of dogs with aldosterone deficiency.

Hypercalcaemia (up to 30% of cases) has been described in hypoadrenocorticism. The mechanism is poorly understood but decreased glomerular filtration rate, metabolic acidosis, reduced calciuresis, increased vitamin D activity and increased intestinal calcium absorption have all been proposed. The contribution of metabolic acidosis to mild ionized hypercalcaemia is supported by the inverse relationship between venous pH and ionized calcium concentration in dogs with hypoadrenocorticism, likely reflecting a reduction in binding to plasma proteins [26]. In a small study of eight hypercalcaemic dogs with hypoadrenocorticism, parathyroid hormone (PTH), parathyroid hormone-related protein (PTHrp) and 1,25 dihydroxyvitamin D (1,25 (OH)_2_ D) concentrations were within or below reference interval, making hyperparathyroidism, hypercalcaemia of malignancy or hypervitaminosis D unlikely [58]. Given the relatively high prevalence of hypercalcaemia in dogs with hypoadrenocorticism, adrenal function tests should be performed if this electrolyte abnormality is observed, particularly when a neoplastic cause is not readily apparent.

Increased urea (up to 82% of cases), creatinine (up to 66% of cases) and phosphate (up to 58% of cases) concentrations commonly occur in hypoadrenocorticism due to the decreased glomerular filtration rate associated with dehydration and hypovolaemia. Whilst the azotaemia associated with hypoadrenocorticism is typically pre-renal, differentiation from renal causes is complicated by the often decreased urine specific gravity associated with aldosterone deficiency and sodium wastage [59]. Increased urea concentrations can also reflect gastrointestinal haemorrhage and therefore can be seen with glucocorticoid deficiency alone (approximately 15% of cases) [29].

Other biochemical abnormalities associated with hypoadrenocorticism include hypocholesterolaemia, hypoglycaemia and hypoalbuminaemia. Hypocholesterolaemia is suspected to relate to deficits in gastrointestinal fat absorption and is supported by development of glucocorticoid-responsive steatorrhea in adrenalectomized humans and rats [60]. Hypocholesterolaemia is more common in dogs with glucocorticoid deficiency alone approximately 75 versus 15–20% cases, respectively) [14, 28, 29]. Indeed, hypoadrenocorticism was the third and fourth most common cause of moderate to severe hypocholesterolaemia in dogs after hepatic and gastrointestinal disease in two retrospective studies [61, 62]. Hypocholesterolaemia is likely a consequence of the relatively prolonged gastrointestinal signs in dogs with this form of the disease. Hypoglycaemia has been reported in up to one third of cases of hypoadrenocorticism and appears just as likely with glucocorticoid deficiency alone [18, 25]. Glucocorticoid deficiency leads to decreased hepatic gluconeogenesis and increased peripheral sensitivity to insulin which can result in hypoglycaemia.

Hypoalbuminaemia is also common in dogs with hypoadrenocorticism. Causes are multifactorial and include impaired synthesis, lack of nutrient intake from anorexia, impaired absorption of nutrients, gastrointestinal loss and haemorrhage. As with hypocholesterolaemia, hypoalbuminaemia is more commonly reported in dogs with glucocorticoid deficiency alone likely reflecting chronicity and predominance of gastrointestinal signs. [28, 29]. Mild to moderate increases in ALT and AST activities have been described (30% of cases), possibly due to decreased hepatic perfusion.

Metabolic acidosis is present in approximately two thirds of affected dogs [41]. It was traditionally attributed to mineralocorticoid deficiency related impaired renal hydrogen ion secretion along with hypovolaemia and hypotension that result in lactic acidosis. Concurrent hyperkalaemia also impairs synthesis and excretion of ammonium anions, which further exacerbates metabolic acidosis. A recent study in which a semiquantitative approach to acid base analysis was performed in addisonian dogs identified that metabolic acidosis is mostly due to the free water loss secondary to hyponatraemia. In addition, hyperphosphataemia and unmeasured anions such as sulphates, urate and hypurates are contributing factors, whilst lactic acidosis was relatively uncommon [63].

Concurrent hypothyroidism has been diagnosed in several cases of hypoadrenocorticism [41]. Care must be taken when diagnosing hypothyroidism with concurrent hypocortisolism as total thyroxine concentrations can be reduced in dogs with non-thyroidal illnesses and TSH concentrations can be increased in approximately 30% of dogs with primary hypoadrenocorticism [64]. As a result, the diagnosis of hypothyroidism can be challenging and is best investigated after stabilisation of the hypoadrenocorticism, although reversal of these changes can take up to 4 months after starting glucocorticoid supplementation.

Recently, the diagnostic value of machine learning methods using boosted tree algorithms has been assessed using routinely collected haematological and biochemical parameters. A model was developed with a sensitivity of 96.3% and specificity of 97.2% (area under the receiver operator characteristic curve of 0.994) for diagnosis of hypoadrenocorticism. Whilst outperforming the diagnostic capabilities each of lymphocyte count, Na:K ratio and a regression model using lymphocyte count and Na:K ratio, it was not significantly different to basal cortisol analysis. Additionally, these algorithms were evaluated in a referral population of dogs in which hypoadrenocorticism was clinically suspected, and further validation is required in a wider population of dogs [65].

**Table 2:** The prevalence of individual routine clinicopathological features of both hypoadrenocorticism and ‘atypical’ hypoadrenocorticism compiling data from the most relevant case series [14, 18, 25, 26, 28, 29, 37, 38, 42, 46, 66]. In most cases of primary hypoadrenocorticism, mineralocorticoid deficiency was presumed because of associated electrolyte abnormalities. In most cases of ‘atypical’ hypoadrenocorticism, electrolyte abnormalities were not present and while glucocorticoid deficiency was confirmed, mineralocorticoid production was not usually evaluated. Secondary hypoadrenocorticism was confirmed in only a small number of these cases but included two cases with hyponatraemia

Abnormality	Hypoadrenocorticism		“Atypical” hypoadrenocorticism	
	No. affected (No. evaluated)	%	No. affected (No. evaluated)	%
**Haematology**				
Anaemia	130 (507)	27.0	23 (68)	33.8
Neutrophilia	82 (334)	24.5	16 (68)	23.5
Eosinophilia	81 (441)	18.4	13 (66)	19.7
Relative erythrocytosis	64 (369)	17.3	0 (68)	0
Lymphocytosis	38 (367)	10.4	14 (68)	20.6
**Biochemistry**				
Hyperkalaemia	467 (535)	87.2	0 (78)	0
Increased urea	386 (472)	81.7	11 (67)	16.4
Hyponatraemia	427 (533)	80.1	2 (78)	2.6
Increased creatinine	241 (363)	66.4	4 (18)	22.2
Hyperphosphataemia	273 (468)	58.3	0 (68)	0
Increased ALT	85 (257)	33.1	14 (65)	21.5
Increased AST	83 (260)	31.9	16 (41)	39
Increased ALP	79 (260)	30.4	6 (65)	9.2
Hypercalcaemia	144 (492)	29.3	1 (18)	5.5
Hypoglycaemia	88 (553)	15.9	21 (67)	31.3
Hypocholesterolaemia	42 (322)	13.0	45 (62)	72.5
Hypoalbuminaemia	37 (359)	10.3	49 (66)	74.2
**Urinalysis (hypoadrenocorticism only)**	**Mean**	**Range**	**Azotaemia and USG < 1.030**	**%**
Urine specific gravity	1.023	1.004–1.055	112 (193)	58

*No.* Number; *ALT* Alanine aminotransferase; *ALP* Alkaline phosphatase; *AST* Aspartate aminotransferase; *USG* Urine specific gravity

**Table 3:** Diagnostic performance of different clinicopathological, imaging, and endocrine abnormalities described in dogs with hypoadrenocorticism

Study	Parameter	Cut-offs	No./type of cases	Sensitivity % (CI)	Specificity & (CI)
**Seth et al. 2011**	Lymphocyte count	> 5.0 × 10^9^/L	53 HA 110 DMH	19 (9–32)	99 (95–100)
**Zeugswetter et al. 2014**	Lymphocyte count	> 2.45 × 10^9^L	38 HA 107 DMH	92	91
**Zeugswetter et al. 2014**	Neutrophil:lymphocyte	≤ 2.3	38 HA 107 DMH	67	82
**Zeugswetter et al. 2014**	Eosinophil count	> 3.98 × 10^9^/L	38 HA 107 DMH	91	72
**Seth et al. 2011**	Na:K	< 27	53 HA 110 DMH	70 (56–82)	94 (87–97)
		< 24		62 (48–75)	96 (91–99)
		< 20		51 (37–65)	100 (97–100)
**Zeugswetter et al. 2014**		≤ 22	38 HA 107 DMH	92	91
**Adler et al. 2007**		< 27	76 HA 200 HP	89 (80–95)	97 (93–99)
		< 24		79 (67–86)	100 (97–100)
**Boretti et al. 2015**		≤ 24	23 HA 109 DMH	56 (35–77)	99 (93–100)
**Reagan et al. 2020**	Machine algorithm		133 HA + 908 DMH	96.3 (81.7–99.8)	97.2 (93.7–98.8)
**Wenger et al. 2010**	Right adrenal gland thickness	< 3.05 mm	22 HA, 14 H, 10 NAI	82	90
	Left adrenal gland thickness	< 2.8 mm	29 HA, 14 H, 10 NAI	90	100
**Lennon et al. 2007**	Basal cortisol	≤ 55 nmol/L	13 HA 110 NAI	100	78.2
		≤ 27.59 nmol/L		100	98.2
**Bovens et al. 2014**		≤ 55 nmol/L	14HA 450 NAI	100	63.3
		≤ 27.59 nmol/L		85.7	91.8
**Boretti et al. 2015**		≤ 55 nmol/L	23 HA 109 DMH	100 (85–100)	20 (13–22)
**Gold et al. 2016**		≤ 55 nmol/L	163 HA 359 NAI	99.4	67
		< 22 nmol/L		96.9	95.7
		≤ 5.5 nmol/L		81.6	99.1
**Zeugswetter et al. 2014**	eACTH	> 50 pmol/L	38 HA 107 DMH	96	100
**Boretti et al. 2015**		> 178 pg/mL	23 HA 109 DMH	91 (72–99)	99 (93–100)
**Boretti et al. 2015**	Cortisol to eACTH ratio	≤ 0.01	23 HA 109 DMH	100 (85–100)	99 (93–100)

*eACTH* endogenous adrenocorticotropic hormone; *CI* Confidence interval; *DMH* Diseases mimicking hypoadrenocorticism; *HA* Hypoadrenocorticism; *HP* Hospital population; *H* Healthy; *NAI* Non-adrenal illness; *Na:K* Sodium to potassium ratio

### Diagnostic imaging

As previously discussed, megaoesophagus has been described radiographically in dogs with hypoadrenocorticism [14, 28, 52–54]. Other findings such as microcardia, small cranial lobar pulmonary artery, narrow posterior vena cava or microhepatica have been observed radiographically, reflecting hypovolaemia rather than specifically indicating Addison’s disease [25].

Ultrasonographic evaluation of the adrenal glands might also provide information suggestive of hypoadrenocorticism with most dogs having a measurable reduction in adrenal size. One study compared adrenal size (length and thickness) of 30 dogs with hypoadrenocorticism, 14 healthy dogs and 10 dogs with diseases mimicking hypoadrenocorticism. Adrenal glands could be found in all healthy dogs and those with diseases mimicking hypoadrenocorticism but could not be identified in all dogs with hypoadrenocorticism, especially the right adrenal gland (not identified in more than 24% of cases) [67]. Significant overlap between groups was observed in the length of the adrenal glands, especially between dogs with hypoadrenocorticism and diseases mimicking hypoadrenocorticism. However, this overlap was less common when the maximal thickness (greatest dorsoventral dimension) of the left adrenal gland was evaluated. Identifying a maximal left adrenal gland thickness less than 2.8 mm was highly suggestive of hypoadrenocorticism [67]. However, evaluation of the size of the adrenal glands must be performed with caution, as there are multiple studies describing the effect of body weight on the adrenal gland thickness in healthy dogs [68–70]. Therefore, a single cut-off cannot apply for dogs of all different sizes. Nevertheless, identifying small adrenal glands in a dog with clinical signs and clinicopathological abnormalities suggestive of hypoadrenocorticism should prompt the clinician to perform further adrenocortical function tests.

### Electrocardiography

Electrocardiography can reveal findings consistent with hyperkalaemia (i.e., bradycardia, lack of P waves, wide QRS complexes and tall T waves) [41]. Hyperkalaemia results in a decrease in the resting membrane potential. In theory, this should result in increased membrane excitability. However, persistent depolarization inactivates sodium channels in the cell membrane leading to decreased excitability, muscle weakness and abnormal cardiac conduction. Hyponatraemia, metabolic acidosis and a rapid change in potassium concentrations can enhance the effect of hyperkalaemia. Conversely, hypercalcaemia reduces the cardiac effects of hyperkalaemia by increasing membrane stability. As a result, electrocardiographic changes cannot be used to estimate the severity of hyperkalaemia and may not be present even with severe hyperkalaemia.

### Adrenocortical function tests

As previously discussed, the history, clinical features and clinicopathological abnormalities should raise the suspicion for hypoadrenocorticism. However, to confirm the diagnosis, evaluation of adrenocortical function must be performed. Different diagnostic tests have been described all with inherent advantages and disadvantages. Before performing any adrenocortical function tests, the clinician must rule out the prior administration of systemic or topical corticosteroids, progestins and certain herbal therapies; as these treatments can affect the interpretation of the results either by interacting with the cortisol assay or through inhibition of the adrenal pituitary hypothalamic axis.

#### ACTH stimulation test

The ACTH stimulation test, using tetracosactide, is considered the most appropriate test to confirm hypoadrenocorticism. Samples are taken before and approximately 60 min after ACTH administration. Although doses of 5 ug/kg are typically used, doses as low as 1 ug/kg may be sufficient [71]. In healthy dogs, there is no difference between cortisol concentrations one-hour post-administration of intravenous, subcutaneous or intramuscular synthetic ACTH [72, 73]. However, the absorption of ACTH administered subcutaneously or intramuscularly in severely dehydrated dogs might be delayed affecting the interpretation of the test. As a consequence, intravenous administration is preferred [18, 74].

A diagnosis of hypoadrenocorticism relies on the demonstration of diminished cortisol reserve in the absence of recent corticosteroid administration [75]. If administration of corticosteroids is clinically necessary prior to performing an ACTH stimulation test, dexamethasone should be used as it is the only corticosteroid that does not cross react in cortisol assays. However, treatment with dexamethasone can induce suppression of the adrenal pituitary hypothalamic axis. One study indicated that a single intravenously administered dosage of 0.1 mg/kg of dexamethasone can alter the results of the ACTH stimulation test for up to 3 days in healthy beagles. The duration of suppression of pre-ACTH plasma cortisol concentration after dexamethasone was clearly dose dependent, with a dose of 5 mg/kg causing decreased pre-ACTH plasma cortisol and endogenous ACTH concentrations for at least 3 days. However, although the response was decreased, there was still adequate stimulation to exclude a diagnosis of hypoadrenocorticism. Therefore, complete suppression of response to ACTH after a single dose of dexamethasone would be unlikely [76]. However, longer periods of treatment will result in suppression of the adrenal pituitary hypothalamic axis and a false positive result for hypoadrenocorticism. An inadequate cortisol response to ACTH stimulation can result from prior glucocorticoid administration, treatment with drugs that suppress adrenal function such as ketoconazole, reduced potency of the ACTH due to storage, inappropriate dose of ACTH, errors in ACTH administration or sample collection, non-cortisol secreting adrenal tumours and critical illness related adrenal insufficiency [41].

Post-ACTH cortisol concentrations show minimal stimulation and values below approximately 55 nmol/L have traditionally been considered consistent with hypoadrenocorticism [41]. In most dogs with hypoadrenocorticism, both pre- and post-ACTH cortisol concentrations are much lower. A post-ACTH cortisol concentration above this value (55 nmol/L) but below the value consistent with adequate adrenal reserve (220 nmol/L) is considered equivocal [41]. However, one study demonstrated that dogs with stimulated but equivocal post ACTH cortisol concentrations were unlikely to develop hypoadrenocorticism when followed for a median of 2 years [29]. Consequently, such values are unlikely to indicate current or impending hypoadrenocorticism.

The ACTH stimulation test does have limitations. In some countries, synthetic ACTH is expensive and intermittently unavailable [77]. The test only provides evidence of cortisol deficiency, and results do not distinguish between primary, central or iatrogenic hypoadrenocorticism. In addition, although rarely reported, isolated mineralocorticoid deficiency would not be identified.

#### Basal cortisol measurement

A basal cortisol concentration ≤ 55 nmol/L has been shown to have a sensitivity of 99.4—100% [77–79] for the diagnosis of hypoadrenocorticism. Values > 55 nmol/L have been reported in only 7/751 (< 1%) dogs with hypoadrenocorticism [12, 25, 30, 80–85]. In light of this high sensitivity, basal cortisol concentration is useful as a screening test. Unfortunately, its low specificity (63.3—78.2%) negates its use to confirm a diagnosis [77–79].

One study evaluated the effect of lowering the cut-off using 351 dogs in which hypoadrenocorticism was clinically suspected but eventually excluded [79]. Decreasing the cut-off to ≤ 5.5 nmol/L increased diagnostic specificity to 99.1% but sensitivity was reduced to 81.6%. Additionally, this lower cut-off is below the limit of quantification of most common commercial assays. It is most useful as a screening test to exclude hypoadrenocorticism, particularly when the need for testing is realised after the administration of glucocorticoids, as it can be performed in residual plasma or serum samples submitted to the laboratory for other reasons.

#### Endogenous adrenocorticotropic hormone concentration and cortisol-to-adrenocorticotropic hormone ratio

Although endogenous ACTH measurement is not required as part of the standard investigations for hypoadrenocorticism, it allows differentiation between dogs with primary isolated glucocorticoid deficiency and dogs with central hypoadrenocorticism [18].

Considering the high cost and limited availability of synthetic ACTH in some countries, the cortisol to endogenous ACTH ratio has been investigated as a diagnostic test for primary hypoadrenocorticism [86–88]. These studies described lower basal cortisol concentrations and higher endogenous ACTH concentrations in dogs with hypoadrenocorticism compared to healthy dogs and those with non-adrenal illnesses including those that mimicked hypoadrenocorticism. However, overlap in these parameters was observed between the different groups but such overlap was not observed when the cortisol to endogenous ACTH ratio was calculated. In one prospective study of 23 dogs with hypoadrenocorticism, 79 dogs with diseases mimicking hypoadrenocorticism and 30 healthy dogs, a cortisol to ACTH ratio ≤ 0.01 had a diagnostic sensitivity of 100% and specificity of 99% [88]. Based on this study, the cortisol-ACTH hormone ratio is a useful diagnostic test. Care is required in sampling dogs for endogenous ACTH measurement as it is labile and special sampling and transport criteria need to apply. Additionally, samples must be taken before any corticosteroid administration.

In dogs with central hypoadrenocorticism, low endogenous ACTH concentrations are expected. Similar results are observed with prior corticosteroid/progestin administration.

#### Plasma aldosterone concentrations pre- and post-ACTH stimulation test

In most cases, mineralocorticoid deficiency is usually inferred from abnormal sodium or potassium concentrations, or both, at initial diagnosis and specific measurement is not indicated. However, reference interval electrolyte concentrations are possible in dogs with mineralocorticoid deficiency. In such cases, there is a risk of developing electrolyte disturbances in the future. Aldosterone secretory capacity can be evaluated by measuring plasma aldosterone concentration before and 60 min after administration of synthetic ACTH [15, 89].

Theoretically, assessment of the aldosterone secretory capacity in dogs with hypoadrenocorticism can help in the differentiation of primary and secondary hypoadrenocorticism as well as whether dogs are affected by isolated glucocorticoid deficiency. Both basal plasma aldosterone and ACTH-stimulated aldosterone concentrations are expected to be low in dogs with primary hypoadrenocorticism and hypoaldosteronism and within reference interval in dogs with isolated glucocorticoid deficiency and also secondary hypoadrenocorticism, the latter two disorders requiring differentiation through endogenous ACTH measurement. Basal plasma aldosterone and ACTH-stimulated aldosterone concentrations have been shown to be significantly lower in dogs with hypoadrenocorticism compared to healthy dogs and dogs with non-adrenal illness mimicking hypoadrenocorticism. However, there was overlap between groups for both. Aldosterone concentrations were within reference interval in a minority of dogs with primary hypoadrenocorticism and electrolyte abnormalities and undetectable in others with reference interval electrolyte concentrations [15, 86]. Nevertheless, the results of this test provide information as to the need for mineralocorticoid along with glucocorticoid supplementation in dogs with hypocortisolaemia without electrolyte abnormalities.

#### Plasma aldosterone to renin ratio

This diagnostic test can be performed in cases in which isolated mineralocorticoid deficiency is suspected based on the presence of characteristic electrolyte changes and clinical signs compatible with mineralocorticoid deficiency with exclusion of other possible causes. However, these cases are rarely reported and therefore studies evaluating the performance of this diagnostic test are lacking. Only one study has evaluated aldosterone concentration and renin activity in dogs with hypoadrenocorticism and heathy control dogs. This study identified overlap in aldosterone concentration and renin activity between groups. However, the aldosterone to renin ratio was shown to be significantly lower in dogs with hypoadrenocorticism compared to healthy dogs [86]. These results are promising, although this test requires further investigation, notably in dogs with non-adrenal illness. Unfortunately, sample handling requirements, limited assay availability and cost currently prevents the routine evaluation of renin activity in clinical practice.

## Conclusion

Hypoadrenocorticism in dogs is a relatively uncommon disease that can present with a wide variety of clinical signs, resulting from glucocorticoid or mineralocorticoid deficiency, or both. These dogs can present with vague, waxing and waning clinical signs (mainly gastrointestinal) related to glucocorticoid deficiency or with more severe, even life-threatening illness with hypovolaemic shock, collapse and severe hyperkalaemia related to mineralocorticoid deficiency. Hypoadrenocorticism should be considered in all dogs presenting with severe illness and typical electrolyte abnormalities but also in those with chronic waxing and waning signs even with reference interval electrolyte concentrations. The ACTH stimulation test is considered the test of choice for diagnosing hypoadrenocorticism and should be performed in all dogs with clinical suspicion of hypoadrenocorticism. Evaluation of mineralocorticoid reserve capacity at the same time will help depict those with mineralocorticoid deficiency that do not have typical electrolyte changes. If mineralocorticoid production is sufficient, endogenous ACTH measurement may provide a means of distinguishing primary from secondary disease.

## Data Availability

The data discussed in this review article is published and cited.

## Abbreviations

ACTH: Adrenocorticotropic hormone
ALIVE: Agreeing language in veterinary endocrinology
ALT: Alanine aminotransferase
AST: Aspartate aminotransferase
AVP: Arginine vasopressin
CRH: Corticotropin releasing hormone
ESVE: European society of veterinary endocrinology
PTH: Parathyroid hormone
PTH-rp: Parathyroid hormone related proteins
TSH: Thyroid stimulating hormone
